# Modeling the risk of malaria for travelers to areas with stable malaria transmission

**DOI:** 10.1186/1475-2875-8-296

**Published:** 2009-12-16

**Authors:** Eduardo Massad, Ronald H Behrens, Marcelo N Burattini, Francisco AB Coutinho

**Affiliations:** 1 Department of Pathology, School of Medicine, University of Sao Paulo and LIM 01-HCFMUSP, Rua Teodoro Sampaio, 115, CEP: 05405-000 - São Paulo, S.P., Brazil; 2 Department of Epidemiology and Population Health, London School of Hygiene and Tropical Diseases, UK

## Abstract

**Background:**

Malaria is an important threat to travelers visiting endemic regions. The risk of acquiring malaria is complex and a number of factors including transmission intensity, duration of exposure, season of the year and use of chemoprophylaxis have to be taken into account estimating risk.

**Materials and methods:**

**A **mathematical model was developed to estimate the risk of non-immune individual acquiring falciparum malaria when traveling to the Amazon region of Brazil. The risk of malaria infection to travelers was calculated as a function of duration of exposure and season of arrival.

**Results:**

The results suggest significant variation of risk for non-immune travelers depending on arrival season, duration of the visit and transmission intensity. The calculated risk for visitors staying longer than 4 months during peak transmission was 0.5% per visit.

**Conclusions:**

Risk estimates based on mathematical modeling based on accurate data can be a valuable tool in assessing risk/benefits and cost/benefits when deciding on the value of interventions for travelers to malaria endemic regions.

## Introduction

The risk of malaria for visitors to the nine Brazilian states of the Legal Amazon region - Acre, Amapá, Amazonas, Maranhão (western part), Mato Grosso (northern part), Pará (except Belém City), Rondônia, Roraima and Tocantins (western part) - is predominantly P. vivax (75%) with P. falciparum making up the remainder one quarter of surveillance reports. In addition, it should be noted that multidrug-resistant P. falciparum has been reported [1] in the same region. Transmission occurs in most forested areas below 900 m though there is some urban transmission around settlements and small cities in the region. Transmission intensity varies according to the season and municipality. It is higher in jungle areas where recent (<5 years) mining, lumbering and agricultural settlements than in urban areas, such as larger cities like Boa Vista, Macapá, Manaus, Maraba, Pôrto Velho, Rio Branco and Santarém, where transmission occurs on their outskirts. However, in the central areas of these cities transmission is negligible or non-existent.

In 2007 Brazil reported approximately 50% of the total number of the malaria cases in the Americas. Ninety-nine percent of those cases were from the Legal Amazon, where 10% to 15% of the population of Brazil population live [2]. Case numbers fell between 1992 to 2002 from 572,000 to 349,873, with around 16.5% of all the slides examined resulted positive for malaria. A rebound occurred between 2003 to 2007 with number of cases peaking at 607,000 in 2005 and 458,041 cases in 2007. All reported malaria cases were confirmed by laboratory analysis, and 19% in 2007 were *P. falciparum*. Theses cases were predominantly associated with population movement to the periphery of large cities in the Legal Amazon Region [2]. Therefore, the average burden of malaria over the last decade has been approximately 600,000 cases per year, with the proportion of falciparum around 20% ot total [3]. WHO estimated the total numbers of malaria cases in 2006 as approximately 1.4 million [2]. The difference between the two figures reflects either an underestimation (Brazilian official data) or an overestimation of the actual number of cases (WHO estimates). The true values probably lies between the two.

Brazil has the second largest number of foreign visitors in Latin America after Mexico [4]. In 2005 Brazil recorded 5.4 million international arrivals with 57% of these traveling coming from North America and Europe [5]. Of the total, 44% were leisure tourists. Preliminary analysis of tourism arrivals for 2004/2005 by Embratur [5], reveal that 39% of tourists cite Brazil's natural beauty as their reason for travel. However, 7% of leisure tourists ((3% of total tourists) state they visited the Brazilian Amazon. Therefore, estimated visits to the malaria endemic areas of Brazil are of the order of 160,000 per year. Embratur [5] identifies that tourists from the domestic market is much larger. The latest study indicates that of the annual 11 million domestic Brazilian travelers, around 300,000 visit the Amazon region. Therefore an estimated half a million non-resident visitors are exposed to malaria per year in this region [4].

Malaria prevention in non-immune travelers is based on chemoprophylaxis, recommended for all visitors to the region where there is active malaria transmission. However, all regimens have well recognized and not infrequent side effects, including severe events that interfere with routine daily activity. Therefore risk-management requires the balance of risk of infection and risk of toxicity when prescribing chemoprophylaxis. This balance is particularly important when the risk of malaria is low and the numbers exposed are significant [6].

This study was designed to use a mathematical model to estimate the risk of acquiring falciparum malaria for travelers to the endemic regions of Brazil.

## The Model

The model assumes that the population of humans is subdivided into three classes and the population of mosquitoes is similarly divided into three compartments summarized in table S1, Additional file 1. It was separated from the human general population (individuals that are in the area) a cohort [7], denoted by primes and named "probe", which represent a cohort of travelers, followed through their entire exposure in the region, to calculate the risk of malaria acquisition.

The model's dynamics is described by the set of equations shown in the appendix.

A deterministic version (precisely determined through a known relationship) of the model was used to describe the malaria dynamics in the resident population level and a stochastic version (using a ranges of variable values providing a probability). On the equations analyzing the probes to describe the risk (probability of contracting malaria) of a single individual traveler visiting the region. This is based on the assumption that, since the probe is a small number of individuals, the biting rate will randomly fluctuate and the probability of infection is unpredictable.

The model's parameters are: *a *is the mosquitoes daily biting rate; *a*' is the mosquitoes daily biting rate in the probe; *b *is the proportion of infected bites that are actually infective to humans; *b*' is the proportion of infected bites that are infective to humans in the probe; *c *is the proportion of bites that are infective for mosquitoes; *μ*_*H *_is the humans mortality rate; *γ*_*H *_is the humans recovery rate from parasitaemia; *r*_*H *_is the humans birth rate; *α*_*H *_is the malaria-induced mortality rate of humans; *σ*_*H *_is the lost of immunity due to malaria; *μ*_*M *_is the mosquitoes daily mortality rate, *τ *is the extrinsic incubation period; *r*_*M *_is the mosquitoes fertility rate; *κ*_*H *_is the humans carrying capacity and *κ*_*M *_is the mosquitoes carrying capacity. We introduced the term [*c*_*S*_-*d*_*S*_*sin*(2*πft*)] in the susceptible mosquitoes population in order to simulate seasonality in the mosquitoes population [8,9]. The parameters *c*_*S *_and *d*_*S *_(*c*_*S *_>*d*_*S*_) vary the intensity by seasonality, mimicking severe or mild winters, through adjusting these parameters' values. The model parameters are shown in table S2, Additional file 2.

The seasonality parameters *c*_*S *_and *d*_*S *_where chosen to represent the observed seasonal variation in the Amazon region described by Tadei [10], who described a 30 fold difference in mosquito number between summer and winter.

Using the parameters in table S1, Additional file 1 the model calculates that around 250,000, falciparum malaria cases will occur annually, a number similar to WHO estimates for Brazil in 2006 [2].

## Estimating the risk of malaria

In order to calculate the probability of an individual acquiring malaria infection, *π*_*mal *_after the introduction of a single case in an entirely susceptible population it was considered the probe (travelers within the region) followed through an entire outbreak. The probability of infection in this self-limiting outbreak is then given by the following expression:(1)

In the above equation, *S*'_*H*_(*t*) and *N*'_*H*_(*t*) are respectively the number of susceptible hosts and the total population of the cohort used as a probe, and *h*_*mal*_(*t*) is the force of infection of malaria, defined as the per capita number of new cases per time unit [7] and expressed as(2)

where *I*_*M*_(*t*) is the number of infected mosquitoes.

One can also calculate the average risk (probability) of infection for a traveler who arrives in the affected region at week Ω after the outbreak is triggered and remains there for *ω *weeks, . This is done by setting the limits of integration in equation (1) as:(3)

**Figure 1:**
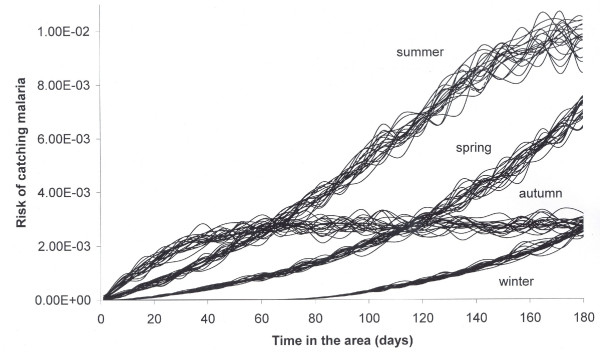
**Stochastic risk of catching falciparum malaria for travelers as a function of the period of the year they arrive and the time remaining in the area (the figure shows 20 iterations of the 1000 simulated)**.

The average risk for a traveler who arrived in the Amazonian region at four different time periods was calculated, namely, in the dry season (winter) in the spring, in the wet season (summer) and in the fall. The model produces a result of 250,000 cases falciparum malaria per year. This number is very dependent on a number of other variables and parameters. In the sensitivity analysis below all the parameters are varied and as a consequence the yearly number of cases varies. The result for the risk calculation is shown in figure 1.

### Sensitivity analysis

In this section we analyze the sensitivity of the model to the parameters. This is done in two steps: a deterministic analysis at the populational level, which describes the sensitivity of the model to measurement variance in the parameters; and a stochastic analysis at the individual level, which determines the variation in the model's outcomes due to intrinsic stochasticity in some of the parameters.

### Sensitivity of the model to variance in the parameters measures

The risk of malaria acquisition *π *as given by equation (3) is a function of a number of parameters collectively denoted by *Par*_*i*_. For a small variation of *Par*_*i*_, Δ*Par*_*i*_, the variation in the risk *π*, Δ*π*, is given by the well-known error-propagation formula [11]:(4)

The relative variation in the risk *π*, Δ*π*/*π*, as a function of the relative variation in the parameters Δ*Par*_*i*_/*Par*_*i*_, is therefore:(5)

The result of this analysis is given in table S3, Additional file 3.

The sensitivity of the model is significantly influenced by with the season of the year. The two parameters that are most influential in the model i are the biting rate *a *and the mosquitoes mortality rate *μ*_*M*_. Biting rate and mosquito mortality are well recognized by entomologists as important parameters as they describe vectorial capacity (a quadratic component) and mortality expressed exponentially in the equation.

### Variation in the model's outcomes due to intrinsic stochasticity in some of the parameters

Of all the parameters described in table S1, Additional file 1 the biting rate a single individual is subject, *a'*, and the probability that an infectious biting is infective to the individual, *b'*, for an occasional traveler are obviously stochastic variables. As mentioned above, it was assumed a Poisson distribution for the parameter *a' *and a Gamma distribution for the parameter *b' *with a small variance. The result is shown in table S4, Additional file 4 which shows the risk of malaria acquisition for a traveler who arrives at different moments of the year and remains 30 days in the area.

**Figure 2:**
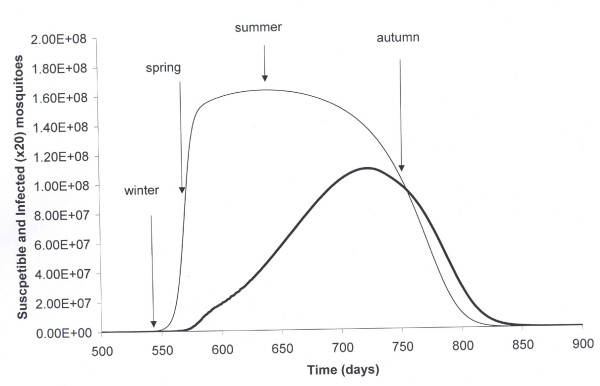
**In the figure we show in the x-axis time in days**. It starts arbitrarily at day 500 and illustrates seasonal variation of a mosquito population (susceptible and infected). The arrows define the relative season and seasonal impact on the susceptible mosquitoes populations (thin line) over the year. The figure also shows the infected mosquitoes (thick line) amplified 20 times.

The highest risk of malaria acquisition occurs for individuals arriving in autumn (around one case for every 500 visitors) as the infected mosquitoes population is close to its peak and the proportion infected high (see figure 2).

Where an individual remains for a 1 year their risk is approximately 1.10 × 10^-2 ^± 2.75 × 10^-5^, that is, a relative error of ± 0.25%. This rate closely correlates with the incidence observed in Amazon residents of 1.16 × 10^-2 ^per person-year. PAHO [12] estimates that this incidence, a maximum of 75 malaria cases per 1000 inhabitants annually will occur. We are estimating only the cases of falciparum malaria, which represent about one third of the total malaria cases, hereby total predicted P falciaprum malaria cases for this region is of the order of 33 cases per 1000 inhabitants per year.

## Discussion

Mathematical models for estimating risks, as described in this paper, should be considered as auxiliary tools for decision-makers. Some caveats, however, are necessary; the model's outcomes are determined by assumptions in the dynamics of the system modeled and on the values given to the parameters. In our model, the most critical assumptions relate to homogeneity. For example, the Amazon region is very large and therefore, it is likely that some parameters will vary from region to region, such as the densities of vectors and human hosts (determined by the respective carrying capacities). The seasonal variations assumed in the model are simple and are only an approximation of the actual climatic variations that occur in the Amazon region. Notwithstanding the oversimplifications of our model, we believe that our results are a good approximation in the sense that the actual risk of malaria lies within the estimated confidence intervals calculated by the model.

Previous studies have attempted to determine the cumulative risk of acquiring malaria in travelers [6,13,14], but the estimated incidence rates were not generalizable to all travelers at all times, as malaria incidence varies greatly from year to year [15].

Mathematical modeling is well suited to adjust for seasonality and annual variations. In a previous analysis we modeled the risk of dengue and yellow fever, with similar approach to the one described here [16-18]. This is the first time that travelers' malaria risk estimates have been calculated using mathematical modeling. Our models are robust and have been tested extensively on Amazonian data [19]. Risk for malaria risk for endemic populations has also been estimated using modeling by Okell et.al [20].

The analysis presented quantifies the risk for non-immune travelers visiting the Amazonian region, adjusting by season and/or epidemic cycle.

A traveler arriving in summer (Dec-Feb) exposed for 120 days has at least a ten-fold higher risk of infection than a traveler who arrives in the winter (June-Aug) for a visit of similar duration. It is shown that the risk increases nonlinearly with time, but this again varies by season of exposure.

National and international recommendations for long term travelers; particularly those traveling through regions of varying transmission and with different malaria species have been very crude (all or nothing) and have a very limited evidence base [21].

The model can be used for highlighting the malaria risk in a way that many advisors and their clients can interpret. An individual arriving during the summer (Jan-Mar), that is, rainy season, has a probability of 0.00015 (1:6666) of being infected within a week of arrival if totally unprotected whilst it takes approximately 3 months for a traveler arriving during the winter months (Jun-Aug), that is, dry season, to be infected with the same likelihood, with intermediary values for arrivals during other seasons. In fact, Behrens et al. [22] estimated the incidence of malaria in UK travelers to Brazil as 1 case per 3000 person-years exposed over the years 2000-2005. During this period there were 394,559 visits with average visit duration of 22.5 days resulting in 9 vivax cases and no P falciparum cases in UK travelers. Running the model with this data the model produced a result of 1 case of falciparum malaria. Assuming that travel was predominantly during the winter months, this single case is a similar incidence (0) as observed in UK travelers, affirming the reliability of our current assumptions and values used in the model.

The model does not take account of pre-existing malaria immunity although for most naïve travelers this is not important. Another aspect that was not considered in this paper is chemoprophylaxis, which is about 95% efficient against falciparum malaria. Therefore, if only 50% of the travelers are compliant, then the number of expected cases is reduced by approximately 48%, although the risk for a non-treated individual does not change.

It is important to stress what is gained in terms of risk estimation with our model. It is known that the annual incidence of malaria among Amazon residents is of the order of 50 cases per 1000 inhabitants. This figure can be used as a proxy for the risk to travelers staying for at least one year in the region. However, the model provides estimations of the risk for travels of shorter durations and, since the risk for these short visits is dependent on the season of the year travelers arrive in the area, the model is essential for those estimations.

The model for the resident population is a modification of the classical Macdonald model [23]. The sensitivity of the model's outcome for errors in the measurements of the parameters was calculated. With exceptions of Macdonald [23] and Burattini et al. [8] who analyzed the sensitivity of the basic reproduction number to variation on the parameters, it seems to us that this is the first time the sensitivity of other Macdonald's model outcomes is analyzed. The results of this analysis point to a model that is very sensitive to the mosquitoes biting rate, *a*, and natural mortality rate, *μ*_*M*_. A 1% error in the measurement of these parameters assumed but perhaps this can be improved. The travelers' population was approximately treated stochastically. By this we mean that we considered the bites received by a single individual are Poisson distributed with average equals to the deterministic value of mosquitoes biting rate, *a*, suffered by the resident population. In fact, the number of bites suffered by each individual is the product of the mosquitoes biting rate *a *times the number of infected mosquitoes in a certain area corresponding to the mosquitoes flying range, which was considered to be approximately constant. We also considered that the probability of infection to humans, *b*, as Gamma distributed around the average value used for the resident population and with a small variance.

The basic model could be applied to other regions where local information on force of transmission, parasite rates or similar malariometric data are available. Such risk estimates would help the travel medicine provider with a better starting point in their risk assessment and provide travelers with a feel for what their malaria risk is and balance this with the appropriateness of chemoprophylaxis.

## Appendix 1

The equations describing the model are given below. The symbols describing the populations involved, the parameters and their values are described in the main text.(A1)

The evolution equations for the probe cohort are:(A2)

for(A3)

and *θ*(*t *- *t*_0_) is the Heaviside function.

## Competing interests

The authors declare that they have no competing interests.

## Authors' contributions

EM and FABC designed the study and the model and prepared the first draft.

EM, RHB, FABC and MNB analyzed the results. All authors contributed to the interpretation of the results and agreed the final draft.

## Supplementary Material

Additional file 1 **Table S1**. Models' variables. Click here for file

Additional file 2 **Table S2**. Model's parameters. Click here for file

Additional file 3 **Table S3**. Sensitivity of the model to each of the parameters (Par) in different periods of the year. The analysis assumes a 1% variation in the value of each parameter and the risk was calculated for 30 days of permanence. The values of the parameters are given in table S2. Click here for file

Additional file 4 **Table S4**. Average risk of malaria acquisition (with confidence intervals) for travelers who remain 30 days in the area. Click here for file
