# Supplementary material for: Modeling the risk of malaria for travelers to areas with stable malaria transmission
Source: Malar J. 2009 Dec 16;8:296. doi: 10.1186/1475-2875-8-296 (PMC2806379; doi:10.1186/1475-2875-8-296)
Supplement: Additional file 1 — Table S1. Models' variables. [file 1475-2875-8-296-S1.DOC]

***Table S1. Models’ variables***

|  | |
| --- | --- |
| $S'_{H}$ | Human susceptible individuals in the “probe” |
| $I'_{H}$ | Human infected individuals in the “probe” |
| $R'_{H}$ | Human recovered individuals in the “probe” |
| $S_{H}$ | Human susceptible individuals in the resident population |
| $I_{H}$ | Human infected individuals in the resident population |
| $R_{H}$ | Human recovered individuals in the resident population |
| $S_{M}$ | Susceptible mosquitoes |
| $L_{M}$ | Latent mosquitoes |
| $I_{M}$ | Infected mosquitoes |
