# Supplementary material for: Modeling the risk of malaria for travelers to areas with stable malaria transmission
Source: Malar J. 2009 Dec 16;8:296. doi: 10.1186/1475-2875-8-296 (PMC2806379; doi:10.1186/1475-2875-8-296)
Supplement: Additional file 2 — Table S2. Model's parameters. [file 1475-2875-8-296-S2.DOC]

# *Table S2. Model's parameters*

|  | | | |
| --- | --- | --- | --- |
| Parameter | Biological interpretation | Value | Source |
|  | Mosquitoes' biting rate | $0.3$ $\text{days}^{-1}$ | [24]^*^ |
|  | Mosquitoes' biting rate in the probe | Poisson (0.3) | Assumed |
|  | Probability of infection to humans | $8.8\times10^{-2}$ $\text{days}^{-1}$ | [25] |
|  | Probability of infection to humans in the probe | Gamma (0.088, 0.0003) | Assumed |
|  | Probability of infection to mosquitoes | $8.7\times10^{-2}$ $\text{days}^{-1}$ | Fitted^+^ |
|  | Humans' mortality rate | $3.9\times10^{-5}$ $\text{days}^{-1}$ | [26] |
|  | Recovery rate | $5.5\times10^{-3}$ $\text{days}^{-1}$ | [27] |
|  | Loss of immunity | $3.3\times10^{-2}$ $\text{days}^{-1}$ | [28] |
|  | Malaria's mortality rate | $1.0\times10^{-2}$ $\text{days}^{-1}$ | [29] |
|  | Humans' reproductive rate | $8$ $\text{days}^{-1}$ | [26] |
|  | Humans' carrying capacity |  | [26] |
|  | Mosquitoes' mortality rate | $1.0\times10^{-1}$ $\text{days}^{-1}$ | [30] |
|  | Extrinsic incubation period |  | [27] |
|  | Mosquitoes' reproductive rate | $4$ $\text{days}^{-1}$ | [24] |
|  | Mosquitoes' carrying capacity | $2.0\times10^{8}$ $\text{days}^{-1}$ | Fitted^+^ |
|  | Seasonality factor | $7.0\times10^{-2}$ $\text{days}^{-1}$ | Fitted^+^ |
|  | Seasonality factor | $5.3\times10^{-2}$ $\text{days}^{-1}$ | Fitted^+^ |
|  | Frequency of seasonality | $2.7\times10^{-3}$ $\text{days}^{-1}$ | Fitted^+^ |
| *Estimated as the inverse of the gonothophic cycle duration.  ^+^Fitted to reproduce the observed prevalence of falciparum malaria in the area. | | | |
