# Supplementary material for: Modeling the risk of malaria for travelers to areas with stable malaria transmission
Source: Malar J. 2009 Dec 16;8:296. doi: 10.1186/1475-2875-8-296 (PMC2806379; doi:10.1186/1475-2875-8-296)
Supplement: Additional file 3 — Table S3. Sensitivity of the model to each of the parameters (Par) in different periods of the year. The analysis assumes a 1% variation in the value of each parameter and the risk was calculated for 30 days of permanence. The values of the parameters are given in table S2. [file 1475-2875-8-296-S3.DOC]

*Table S3. Sensitivity of the model to each of the parameters (Par) in different periods of the year. The analysis assumes a 1% variation in the value of each parameter and the risk was calculated for 30 days of permanence. The values of the parameters are given in table 2.*

|  | | | | | | | | |
| --- | --- | --- | --- | --- | --- | --- | --- | --- |
|  | Winter | | Spring | | Summer | | Autumn | |
| π | 0.0000026 | | 0.000265 | | 0.001008 | | 0.001961 | |
| Par | $\frac{\partial\pi}{\partial Par}$ | ± Relative error  (%) | $\frac{\partial\pi}{\partial Par}$ | ± Relative error  (%) | $\frac{\partial\pi}{\partial Par}$ | ± Relative error  (%) | $\frac{\partial\pi}{\partial Par}$ | ± Relative error  (%) |
| $a$ | 0.0000914 | 10.5 | 0.00966 | 10.81 | 0.044383 | 13.21 | 0.114948 | 17.58 |
| $b$ | 0.0001556 | 5.22 | 0.01628 | 5.39 | 0.0753904 | 6.58 | 0.195216 | 8.76 |
| $c$ | 0.0004556 | 5.20 | 0.001642 | 5.39 | 0.0761103 | 6.57 | 0.197145 | 8.75 |
|  | -0.0010232 | 0.015 | -0.13629 | 0.021 | -0.560427 | 0.021 | -1.24269 | 0.025 |
|  | -0.0010051 | 1.92 | -0.131761 | 2.48 | -0.548329 | 2.72 | -1.22159 | 3.11 |
|  | 0.0000072 | 0.091 | 0.000734 | 0.0912 | 0.0.0027967 | 0.0915 | 0.0054288 | 0.0913 |
|  | -0.0010004 | 3.83 | -0.13155 | 4.96 | -0.547397 | 5.43 | -1.21920 | 6.21 |
|  | 0 | 0 | -3.75x10^-09^ | 0.000113 | 0 | 0 | -2.98x10^-8^ | 0.000122 |
|  | 9.98x10^-13^ | 6.09 | 1.04x10^-10^ | 6.27 | -4.83x10^-10^ | 7.67 | -1.17x10^-09^ | 9.57 |
|  | -0.000585 | 22.27 | -0.0463206 | 17.45 | -0.173717 | 17.23 | -0.43966 | 22.41 |
|  | -0.0000012 | 3.34 | -0.000166 | 4.38 | -0.000734 | 5.09 | -0.001826 | 6.51 |
|  | 0.0000045 | 6.99 | 0.00063081 | 9.51 | 0.0018048 | 7.16 | 0.00416124 | 8.48 |
|  | 6.56x10^-14^ | 4.99 | 6.87x10^-12^ | 5.17 | 3.20x10^-11^ | 6.36 | 8.37x10^-11^ | 8.53 |
