# Supplementary material for: Modeling the risk of malaria for travelers to areas with stable malaria transmission
Source: Malar J. 2009 Dec 16;8:296. doi: 10.1186/1475-2875-8-296 (PMC2806379; doi:10.1186/1475-2875-8-296)
Supplement: Additional file 4 — Table S4. Average risk of malaria acquisition (with confidence intervals) for travelers who remain 30 days in the area. [file 1475-2875-8-296-S4.DOC]

Table S4. Average risk of malaria acquisition (with confidence intervals) for travelers who remain 30 days in the area.

|  | | | | |
| --- | --- | --- | --- | --- |
|  | Winter | Spring | Summer | Autumn |
| $\overline{\pi}$ | 2.60x10^-6^ | 2.66x10^-4^ | 1.01x10^-3^ | 1.96x10^-3^ |
| C.I.(95%)^-^ | 1.98x10^-6^ | 2.06x10^-4^ | 7.97x10^-4^ | 1.58x10^-3^ |
| C.I.(95%)^+^ | 3.30x10^-6^ | 6.0x10^-5^ | 1.22x10^-3^ | 2.37x10^-3^ |
| ± Relative error (%) | 0.81 | 0.74 | 0.66 | 0.64 |
